# A systematic review of candidate genes and their relevant pathways for metastasis among adults diagnosed with breast cancer

**DOI:** 10.1186/s13058-024-01914-6

**Published:** 2024-11-26

**Authors:** Gina M. Gehling, Miad Alfaqih, Lisiane Pruinelli, Angela Starkweather, Jennifer R. Dungan

**Affiliations:** 1 College of Nursing, University of Florida, 1225 Center Dr, PO BOX 100197, Gainesville, FL 32610-1097 USA; 2 College of Medicine, University of Florida, Gainesville, FL USA

**Keywords:** Single nucleotide polymorphisms, Genetic variants, Genetic mutations, Breast cancer, Metastasis

## Abstract

**Background:**

Presently incurable, metastatic breast cancer is estimated to occur in as many as 30% of those diagnosed with early-stage breast cancer. Timely and accurate identification of those at risk for developing metastasis using validated biomarkers has the potential to have profound impact on overall survival rates. Our primary goal was to conduct a systematic review and synthesize the existing body of scientific knowledge on the candidate genes and their respective single nucleotide polymorphisms associated with metastasis-related outcomes among patients diagnosed with breast cancer. This knowledge is critical to inform future hypothesis-driven and validation research aimed at enhancing clinical decision-making for breast cancer patients.

**Methods:**

Using PRISMA guidelines, literature searches were conducted on September 13th, 2023, using PubMed and Embase databases. The systematic review protocol was registered with INPLASY (DOI: 10.37766/inplasy2024.8.0014). Covidence software was used to facilitate the screening and article extraction processes. Peer-reviewed articles were selected if authors reported on single nucleotide polymorphisms directly associated with metastasis among adults diagnosed with breast cancer.

**Findings:**

We identified 451 articles after 44 duplicates were removed resulting in 407 articles to be screened for study inclusion. Three reviewers completed the article screening process which resulted in 86 articles meeting the study inclusion criteria. Sampling varied across studies with the majority utilizing a case-control design (*n* = 75, 87.2%), with sample sizes ranging from 23 to 1,017 participants having mean age 50.65 ± 4.50 (min-max: 20–75). The synthesis of this internationally generated evidence revealed that the scientific area on the underlying biological contributions to breast cancer metastasis remains predominantly exploratory in nature (*n* = 74, 86%). Of the 12 studies with reported power analyses, only 9 explicitly stated the power values which ranged from 47.88 to 99%.

**Discussion:**

Understanding the underlying biological mechanisms contributing to metastasis is a critical component for precision oncological therapeutics and treatment approaches. Current evidence investigating the contribution of SNPs to the development of metastasis is characterized by underpowered candidate gene studies. To inform individualized precision health practices and improve breast cancer survival outcomes, future hypothesis-driven research is needed to replicate these associations in larger, more diverse datasets.

**Supplementary Information:**

The online version contains supplementary material available at 10.1186/s13058-024-01914-6.

## Introduction

Breast cancer has recently surpassed lung cancer as the mostly commonly diagnosed cancer [1]. It is estimated to occur in around 2.3 million new cases globally with an estimated 685,00 deaths [1]. Breast cancer-related deaths have been predicted to increase by as much as 50% from 685,000 in 2020 to 1 million in 2040 worldwide [2]. Presently incurable, metastatic breast cancer (MBC) is estimated to occur in as many as 30% of those diagnosed with early-stage breast cancer [3]. Despite decades of significant prevention and early detection efforts, prominent challenges remain towards detecting those with early metastatic spread as well as prompt identification of those most likely to develop metastatic disease [4]. Clinically and scientifically, we lack sensitive biomarkers underlying MBC, despite its prevailing impact on mortality.

Genetically informed clinical decision-making provides an opportunity to improve long-term breast cancer health outcomes. Understanding the underlying biological mechanisms at play in the development of metastasis is a critical component for precision oncological therapeutics and treatment approaches. While it can be difficult to obtain data, one study estimated 10% of research funding (excluding UK research data) was dedicated to studying metastatic disease between Jan 1, 2016 and December 31, 2020 [5, 6]. Moreover, logistical challenges likely hinder the collection of data on metastasis, contributing to a dearth of research in this area. Using tumor biomarkers, timely and accurate identification of those at increased risk for developing metastasis has the potential to improve survival rates associated with MBC.

Synthesis of this body of literature can serve as a guide for future hypothesis-driven research and lead to precision health strategies for informed clinical decision making. Our purpose was to conduct a systematic review and synthesize the existing body of scientific knowledge on the candidate genes and their respective single nucleotide polymorphisms (SNPs) associated with metastasis-related outcomes among patients diagnosed with breast cancer.

## Methods

The systematic review protocol was registered with INPLASY (DOI: 10.37766/inplasy2024.8.0014) [7].

### Search strategy

For this systematic review, we sought to answer the question to: “What SNPs are associated with metastasis-related outcomes among adults diagnosed with breast cancer within the last 5 years?” The reporting of this systematic review followed the 2020 Preferred Reporting Items for Systematic Review and Meta-Analyses (PRISMA) statement [8]. The literature search phase was conducted on September 13th, 2023. The PubMed database was searched using the following string: (((breast cancer[MeSH Terms]) AND (“metasta*“[Title/Abstract] OR “prognosis“[Title/Abstract])) AND (genetic variation[MeSH Terms])) AND (polymorphism, single nucleotide[MeSH Terms]) with the following filters applied: in the last 5 years, Humans, Adolescent: 13–18 years, Adult: 19 + years. We searched the Embase database using the following string: #1 AND ‘human’/de AND ([adult]/lim OR [aged]/lim OR [very elderly]/lim) AND ‘article’/it #1 ‘breast cancer’/exp AND ‘genetic polymorphism’/exp AND ‘metastasis’/exp AND [2018–2023]/py and the following filters applied: 2018–2023. All peer-reviewed articles were selected for further screening if they reported on associations between SNPs and metastasis-related outcomes among individuals diagnosed with breast cancer.

### Selection criteria

The team agreed upon selection criteria prior to the screening process. All peer-reviewed articles were included based on the following criteria: (1) human adults 18 years or older, (2) diagnosis of breast cancer, (3) focus on association of genetic variants and metastasis, (4) peer-reviewed primary literature, and (5) there is a full-text article available in English. Articles were excluded if: (1) it was an animal or plant study, (2) participants were under the age of 18, (3) the study had a drug, therapy, or pharmacogenomics focus, (4) the genetic association tested was indirectly related to metastasis (i.e., underlying biological mechanisms associated with metastasis such as cell proliferation), or (5) was considered grey or secondary literature.

**Fig. 1:**
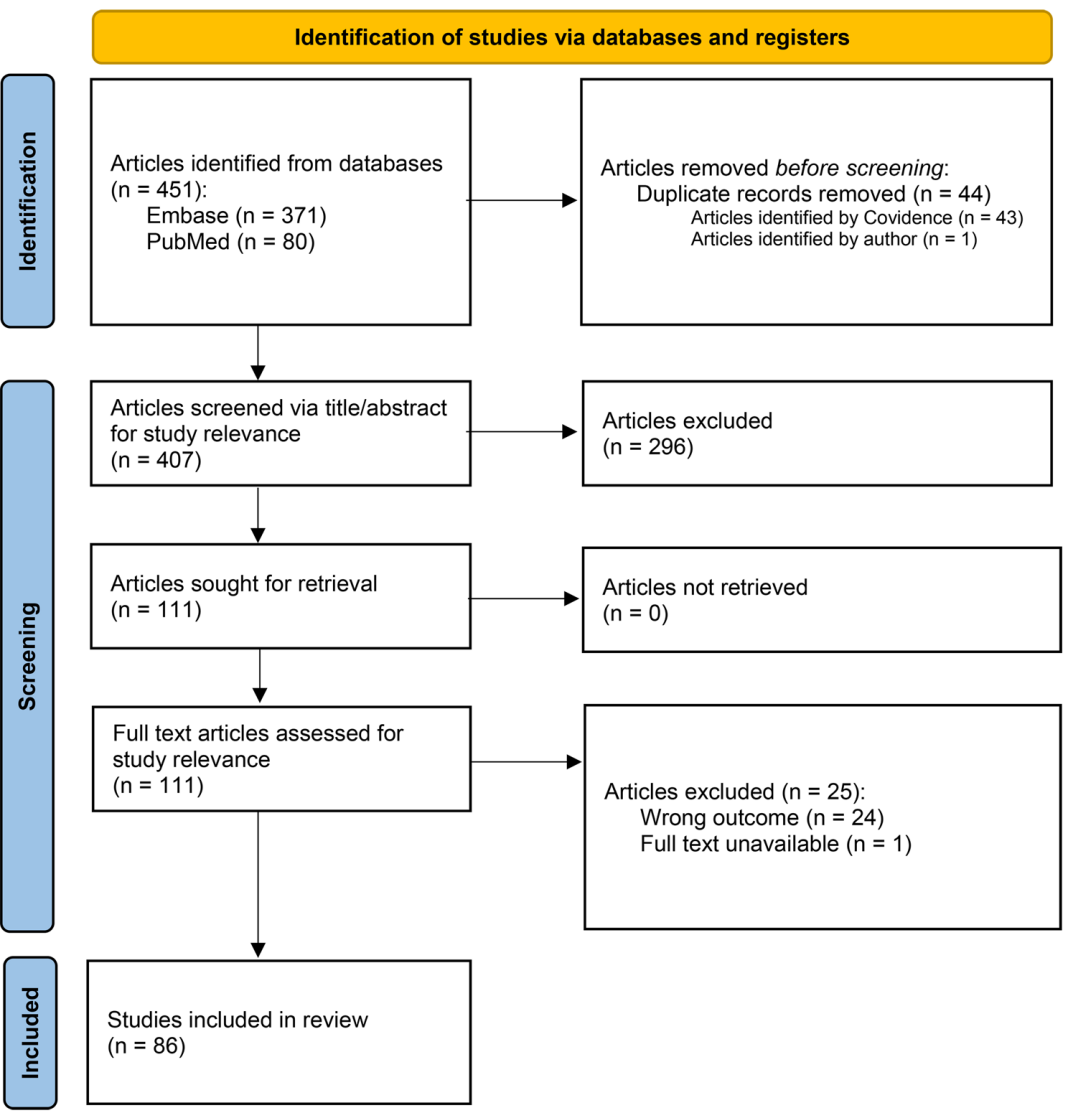
PRISMA flow diagram. Source: Page MJ, et al. BMJ 2021;372:n71. doi: 10.1136/bmj.n71. This work is licensed under CC BY 4.0. To view a copy of this license, visit https://creativecommons.org/licenses/by/4.0/

Using Covidence systematic review software (Veritas Health Innovation, Melbourne, Australia, www.covidence.org*)* to facilitate the screening process, all articles from the search were exported into the software, then three authors (GG, AS, MA) independently screened all articles and met to resolve any conflicts. GG identified 451 articles with 43 duplicates automatically removed by Covidence systematic review software that were then manually verified by GG; whereby GG identified an additional article as being a duplicate for a total of 44 articles marked as duplicates. GG, AS, and MA screen 407 articles during the title/abstract screening phase and deemed 296 articles irrelevant. Of the remaining 111 articles, an additional 25 were excluded during the full text screening phase (wrong outcome *n* = 24 and full text unavailable = 1) resulting in 86 articles included in the review. The PRISMA diagram outlines the flow of the article selection process and can be found in Fig. 1.

### Data extraction

GG used Covidence to develop the data extraction template to facilitate the data extraction process and completion of the literature matrix. Two team members (GG, MA) performed data extraction individually, then met to resolve any conflicts. The full literature matrix was reviewed for accuracy with the following extracted information: select sample characteristics (i.e., sample size, participant age, geographic location), study characteristics (i.e., study design, biological specimen type, type of DNA processing performed—such as genotyping, next generation sequencing, cell culture), list of all investigated genes and their respective SNPs, statistically significant results, whether a power analysis was performed, and a brief summary of findings. Using the extracted data, GG and JD developed an abbreviated literature matrix highlighting studies with statistically significant genetic variants. The matrix is organized by study design and provides details on each SNP’s variant type, chromosomal location, minor allele and minor allele frequency (MAF), the associated metastasis-related outcome, statistical approach, and the corresponding citation. (Supplemental S1) .

## Results

### Sample characteristics

Sampling varied across each study. While all participants were female across all studies, sample sizes ranged widely from 23 to 1,017 cases. For studies that provided sufficient age data, we calculated the mean age of 50.65 ± 4.50 (range: 20–75) years for the case or cohort group (i.e., individuals with histologically and/or pathologically confirmed breast cancer). Of note, there is potential for duplication of participants across studies.

### Study characteristics

A majority of the studies utilized a case control design (*n* = 75, 87.2%) [9–83]. The remaining 11 (12.7%) studies utilized a cohort design [84–94]. Interestingly, 23 countries (Brazil *n* = 3, Turkey *n* = 3, Africa *n* = 5, Iran *n* = 13, Iraq *n* = 1, India *n* = 1, Istanbul *n* = 1, Egypt *n* = 10, Saudi Arabia *n* = 4, China *n* = 21, Lithuania *n* = 3, Pakistan *n* = 1, Sweden *n* = 1, Mexico *n* = 8, Sardinia *n* = 1, Jordan *n* = 2, Poland *n* = 1, United States *n* = 1, Austria *n* = 1, Spain *n* = 1, Sri Lanka *n* = 1, Croatia *n* = 1, and Greece *n* = 1) were represented in this synthesis of the existing literature on the topic of genetic variants in breast cancer and metastasis-related outcomes.

Only 12 studies indicated a power analysis was performed revealing that a majority of this work is exploratory in nature (*n* = 74, 86%). Of those, 9 studies explicitly stated the power values which ranged from 47.4 to 88% [28, 30, 34, 47, 48, 58, 66, 69, 82]. Notably, 3 of the 12 articles that reported a power analysis did not indicate the exact power value [11, 41, 53].

Genomic DNA extracted from blood was the predominant sample specimen type (*n* = 78, 90.7%) used to identify germline SNPs. In contrast, 8 studies investigated somatic mutations in breast metastatic and non-metastatic tissue samples (where reported: tumor tissue *n* = 5; breast/mammary tissue *n* = 4; cancer cell lines *n* = 2; tissue type unspecified *n* = 1) [64, 65, 68, 71, 75, 77, 83, 92].

### Metastasis-related outcomes

**Fig. 2:**
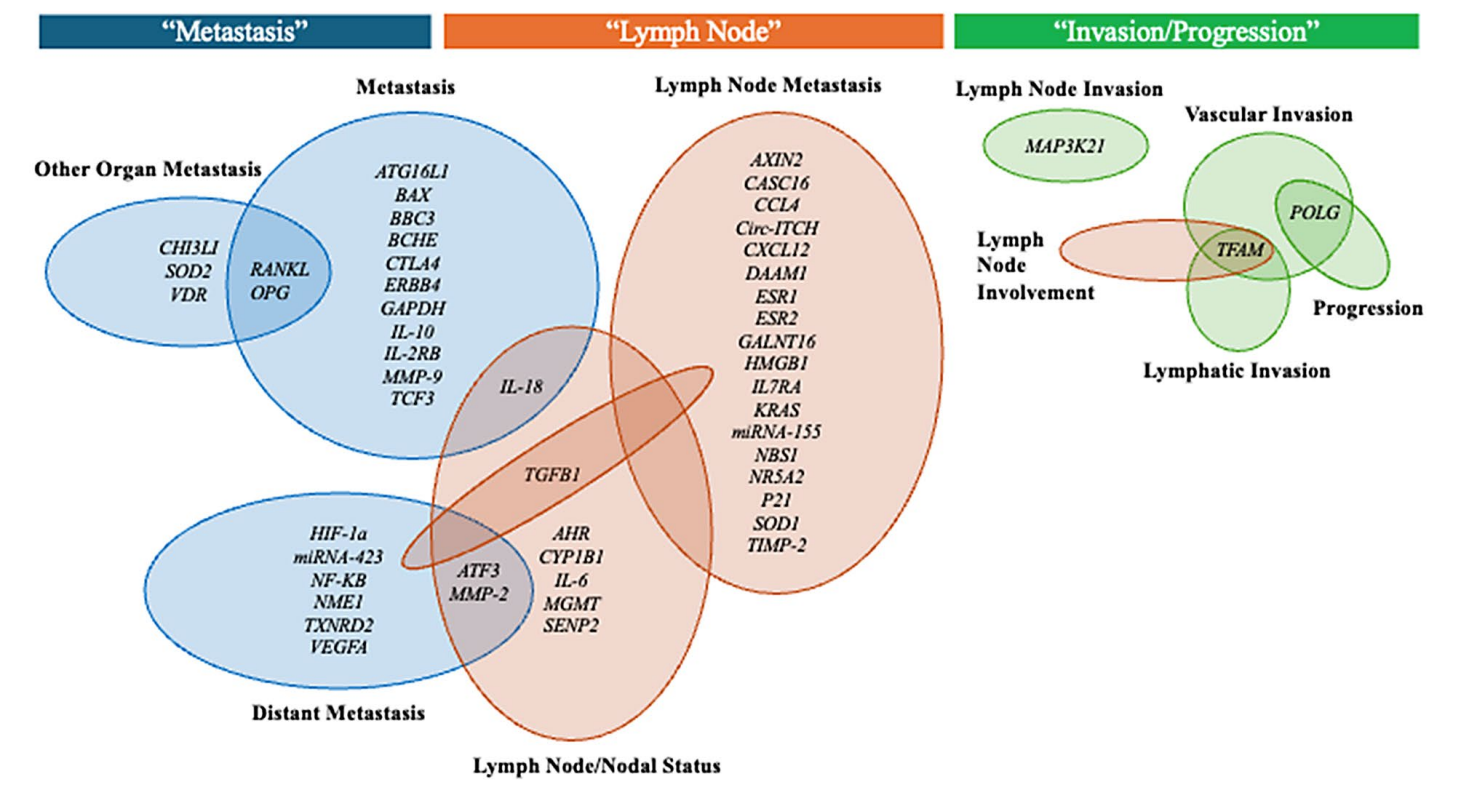
Candidate genes with significant SNPs by BRCA metastasis outcomes. *AHR*^(rs2066853)^ [57]; *ATF3*^(rs3125289, rs11119982)^ [84]; *ATG16L1*^(rs2241880)^ [49]; *AXIN2*^(rs3923087)^ [50]; *BAX*^(rs4645878)^ [14]; *BBC3*^(rs2032809)^ [88]; *BCHE*^(rs1803274)^ [29]; *CASC16*^(rs4784227, rs12922061)^ [39]; *CCL4*^(rs10491121)^ [25]; *CHI3L1*^(rs4950928)^ [42]; *Circ-ITCH*^(rs10485505, rs4911154)^ [73]; *CTLA*^(rs4231775)^ [53]; *CXCL12*^(rs1801157)^ [56]; *CYP1B1*^(rs1056836)^ [57]; *DAAM1*^(rs79036859)^ [71]; *ERBB4*^(rs13423759)^ [46]; *ESR1*^(rs9340799)^ [63]; *ESR2*^(rs1256030)^ [34]; *GALNT16*^(rs2105269)^ [40]; *GAPDH*^(rs1803622)^ [18]; *HIF-1a*^(rs11549465)^ [19]; *HMGB1*^(rs1360485, rs1045411, rs2249825)^ [85]; *IL-10*^(rs1800896)^ [38]; *IL-18*^(rs187238, rs1946518, rs549908)^ [15]; *IL-2RB*^(rs2281089)^ [26]; *IL-6*^(rs1800795, rs1800796, rs1800797)^ [15]; *IL7RA*^(rs6897932)^ [10]; *KRAS*^(rs712, rs61764370)^ [31, 35]; *MAP3K21*^(rs1294255)^ [12]; *MGMT*^(rs12917)^ [57]; *miRNA-155*^(rs767649)^ [24]; *miRNA-423*^(rs6505162)^ [45]; *MMP-2*^(rs2285053, rs243866)^ [28]; *MMP-9*^(rs3918242, rs1056628)^ [13, 20]; *NBS1*^(rs2735383)^ [30]; *NF-KB*^(rs148626207, rs3774937)^ [48]; *NME1*^(rs34214448)^ [54]; *NR5A2*^(rs2246209^^)^ [80]; *OPG*^(rs3102735, rs2073618, rs2073617)^ [27, 42]; *P21*^(rs1801270)^ [84]; *POLG*^(rs2307441, rs2072267)^ [91]; *RANKL*^(rs9533156)^ [27]; *SENP2*^(rs6762208)^ [52]; *SOD1*^(rs2234694, rs4817415)^ [32, 33]; *SOD2*^(rs2758339)^ [62]; *TCF3*^(rs72618599)^ [44]; *TFAM*^(rs3900887, rs11006129)^ [91]; *TGFB1*^(rs1800469, rs1800470)^ [9, 47]; *TIMP-2*^(rs4789936)^ [22]; *TXNRD2*^(rs1139793)^ [84]; *VDR*^(rs2228570, rs1544410)^ [42]; *VEGFA*^(rs833061)^ [55]

During our review of the literature, we discovered metastatic-based prognoses are often made based on several factors including nodal involvement and presence of metastasis. Since we wished to know what genetic variants contribute to metastasis-related outcomes, we included several terms that have a direct relationship to metastasis based on the literature. We identified 53 statistically significant genes (with 72 SNPs) associated with the following metastasis-related outcomes: metastasis (*n* = 13), distant metastasis (*n* = 9), other organ metastasis (*n* = 4), lymph node metastasis (*n* = 19), lymph node involvement (*n* = 1), lymph node/nodal status (*n* = 9), lymph node invasion (*n* = 1), vascular invasion (*n* = 2), lymphatic invasion (*n* = 1), and progression (*n* = 1). Figure 2 provides a visual representation of all the statistically significant candidate genes (*p* < 0.05) by their respective metastasis-related outcomes.

## Discussion

Breast cancer metastasis is considered a major contributing factor to poor prognosis [95]. To our knowledge, our study is the first to report on the state of the science regarding the levels of evidence and study characteristics between SNPs and metastasis-related outcomes among women diagnosed with breast cancer. Currently, the underlying biological and molecular mechanisms associated with the progression of cancer to metastases remains poorly understood. Timely and accurate identification of those at risk for developing metastasis using validated biomarkers has the potential to have a profound impact on overall MBC survival rates.

Alfred Knudson’s two-hit hypothesis states that predisposition to cancer occurs as a result of a dominantly inherited germline mutation and a second “hit” or mutation is required for tumorigenesis [96]. While his theory provided valuable insight into how cancer develops, the process of metastasis is more elaborate with a distinct series of occurrences beyond the initial genetic mutations. The likelihood of further mutations that promote metastasis may be a result of genetic instability attributed to the initial hit in the primary tumor. For a cancer cell to metastasize, it must engage in a complex series of events, collectively known as the metastatic cascade, which extends beyond the initial genetic mutations. Welch and Hurst described the hallmarks of metastasis as the ability of cancer cells to migrate away from the primary tumor (motility), infiltrate through a basement membrane (invasion), restructure local tissue (modulation), react to the modified microenvironment (plasticity), and successfully establish themselves in a tissue (colonization) [97].

Breast cancer is a highly complex and heterogenous disease with several known subtypes that have been shown to have a direct impact on survival outcomes. Breast cancer subtypes investigated among the articles found among this review included luminal A, luminal B, human epidermal growth factor receptor 2 (HER2) +/-, estrogen receptor (ER) +/-, progesterone receptor (PR) +/-, and triple negative breast cancer (TNBC). Considered as one of the most aggressive and challenging to treat breast cancer subtypes, TNBC accounts for roughly 10–15% of all breast cancer diagnoses [98, 99]. Moreover, around 20–30% of early TNBC cases will go on to develop metastasis with a median overall survival (OS) rate for patients with metastatic TNBC estimated at 13.6 months (range 8–13 months) [99]. In contrast, while the 5-year survival rate for women with MBC is around 25% regardless of subtype, those with localized and regional breast cancer have an OS between 86 and 99% [100]. Despite the aggressive nature of TNBC, we were surprised to see Vitiello et al. (2018) was the only article in our study with statistically significant findings among those with this subtype. While these findings are exploratory, their study revealed a genotypic correlation between lymph node metastasis and transforming growth factor beta 1 (*TGFB1*) rs1800460 of 0.29 (*p* < 0.05) and its recessive GCTG haplotype of -0.32 (*p* < 0.05) [9]. They also analyzed the TGFB1 rs1800469 SNP, which showed a recessive model correlation of 0.32 (p < 0.001), and rs1800470, which had a dominant model correlation of -0.31 (p < 0.05) [9]. Additionally, the GTCG haplotype exhibited a recessive model correlation of 0.38 (p < 0.001) [9]. Amongst our study findings, the chemokine ligand 4 (*CCL4*) rs10491121 SNP and the AT genotype compared to the AA genotype among those with the luminal A and luminal B subtypes may play a protective role in reducing risk of developing lymph node metastasis with an odds ratio (OR) of 0.298 and 95% confidence interval (CI) of 0.1-0.885 [25]. Our study also uncovered evidence suggesting that several genes and their respective SNPs may be linked to lymph node metastasis in individuals with the HER2 breast cancer subtype. For example, Gallegos-Arreola et al. (2022) identified the *ESR2* rs1256030 SNP in HER2 cases, with the TT genotype showing an OR of 0.38 (95% CI: 0.18–0.78, *p* = 0.005) [34]. Additionally, Gallegos-Arreola et al. (2021) reported an association between *KRAS* rs61764370 and HER2 histological type breast cancer, with an OR of 3.4 (95% CI: 1.24–9.84, *p* = 0.018) [35]. Vitiello et al. (2018) examined the *IL7RA* rs6897932 SNP in L-HER2-positive cases, finding a Kendall’s tau-b correlation coefficient of 0.32 (additive model, *p* = 0.03) and 0.35 (dominant model, *p* = 0.02) [10].

Our synthesis of this important body of work revealed *TGFB1*, matrix metalloproteinase-2 (*MMP-2*), Nijmegen breakage syndrome protein 1 (*NBS1*), and estrogen receptor 2 (*ESR2*) contain SNPs with statistically significant associations with metastasis-related outcomes from studies with at least 80% power [28, 30, 34, 47, 66]. The *TGFB1* gene has enzyme binding and protein homodimerization activity regulating the growth and cellular differentiation of various cell types, the cellular microenvironment, and the immune response [101]. In the early stages of breast cancer, *TGFB1* functions as a tumor suppressor; however, as the disease advances, its overexpression leads to hyperactivation of the TGFB1 pathway, transitioning its role to that of a tumor promoter thus increasing the risk of cancer development and metastatic progression [102, 103]. The *MMP-2* gene has a vast array of functions including but not limited to vasculature remodeling, tissue repair, tumor invasion, and inflammation [104]. The dysregulation of the MMP-2 pathway is thought to promote a favorable microenvironment for breast cancer proliferation through the degradation of the extracellular matrix thereby promoting cancer cell migration and invasion [105]. While not directly associated with any particular type of cancer, defects in *NBS1*, a gene involved in homologous DNA pairing and strand exchange pathways, may increase cancer risk [104, 106]. Estrogen is a naturally occurring hormone implicated in the growth and function of the reproductive, cardiovascular, nervous, and skeletal system [107]. ESR2-related pathways include gene expression and regulation of phosphatidylinositol 3-kinase (PI3K)/protein kinase B (AKT) signaling [104]. Malfunctioning estrogen receptors result in the dysregulatory function of genes known to play a critical role in cancer prevention by altering normal cellular processes such as degradation, division, and multiplication; the life cycle including apoptosis and longevity; and differentiation [107]. Interestingly, Chi et al. (2019) found that estrogen receptor signaling may undergo changes during breast tumorigenesis [108].

This study is not without its limitations. Identification of true genetic signals in association studies remain challenging. Not to mention variations in racial groups through genetic diversity, environmental exposures, and lifestyle factors likely contribute to epistatic changes and metastatic risks. Our synthesis revealed that the literature represents an array of international studies representing diverse samples, yet only a few of them are adequately powered so definitive causal conclusions cannot be drawn. More diverse, representative populations with sufficient power are necessary for genomic studies to promote optimal generalizability and applicability of results across the globe. Equally important, we acknowledge the inherent limitations associated with this study spanning the time period surrounding the 2020 COVID-19 global pandemic, which has not only impacted the availability of more up to date breast cancer statistics, but has also restricted the advancement of cancer-related research. We recognize that the ideal next step for this study would be a meta-analysis of these findings, however, our evaluation of these studies indicates a great degree of methodological diversity (excess variation in outcomes studied), statistical heterogeneity (inconsistent effects) and diversity in genetic markers tested, along with limited numbers of articles (usually no more than 1 or 2 articles) to meta-analyze [109]. As a result, the feasibility, rigor, and value of meta-analyzing these data are unjustifiable. Lastly, while outside the scope of this particular study, we wish to acknowledge there is emerging research investigating the relationship between SNPs, differential gene expression, and breast cancer-related disease development and progression which will further contribute to our understanding of the biological underpinnings responsible for modulating the development and progression of MBC.

## Conclusion

Explicating the biological drivers of metastasis is a critical component for precision oncological therapeutics and treatment approaches. The results of this systematic review revealed omics predictors of MBC are a nascent field. Furthermore, current evidence investigating the contribution of SNPs to the development of metastasis is characterized by underpowered candidate gene studies. This underscores a clear need for further hypothesis-driven research to fully elucidate the underlying biological mechanisms of breast cancer heterogeneity so that we may better understand its impact on breast cancer metastasis-related outcomes.

## Electronic supplementary material

Below is the link to the electronic supplementary material.

Supplementary Material 1

## Data Availability

The data that support the findings of this study are available upon reasonable request by contacting the corresponding author.

## Abbreviations

ER: Estrogen receptor
ESR2: Estrogen receptor 2
HER: Human epidermal growth factor receptor 2
INPLASY: International Database to Register Systematic Reviews
MMP-2: Matrix metalloproteinase-2
MBC: Metastatic breast cancer
NBS1: Nijmegen breakage syndrome protein 1
PRISMA: Preferred Reporting Items for Systematic Review and Meta-Analyses
PR: Progesterone receptor
SNPs: Single nucleotide polymorphisms
TGFB1: Transforming growth factor beta 1
TNBC: Triple negative breast cancer
