# Supplementary material for: A systematic review of candidate genes and their relevant pathways for metastasis among adults diagnosed with breast cancer
Source: Breast Cancer Res. 2024 Nov 26;26:165. doi: 10.1186/s13058-024-01914-6 (PMC11590482; doi:10.1186/s13058-024-01914-6)
Supplement: Supplementary file 1 — Supplementary Material 1 [file 13058_2024_1914_MOESM1_ESM.docx]

Supplement 1. Statistically significant (p<0.05) breast cancer metastasis-related gene association studies.

| **Source** | **Gene** | **SNP** | **Variant Type** | **Locus** | **Minor Allele** | **Global ALFA**  **MAF** | **Outcome** | **Statistical Approach** | **Individual SNP Result** | **Haplotype SNP Result** |
| --- | --- | --- | --- | --- | --- | --- | --- | --- | --- | --- |
| **Case Control** | | | | | | | | | | |
| Martinez-Ramirez et al., 2021 | *AHR* | RS2066853 | Missense | 7p21.1 | A | 0.12 | Lymph Node Status | OR (95% CI) | G/G: 1 (reference value), p=0.02 | --------------- |
| El-Amir et al., 2020 | *ATG16LI* | RS2241880 | Missense | 2q37.1 | A* | 0.49 | Metastasis | Not reported | AA: p=0.03 | --------------- |
| Dai et al., 2019 | *AXIN2* | RS3923087 | Intron | 17q24.1 | T | 0.27 | Lymph Node Metastasis | OR (95% CI) | TT: 1.83 (1.01-3.32), p=0.030 | --------------- |
| Ozoron et al., 2022 | *BAX* | RS4645878 | 2KB Upstream | 19q13.33 | A | 0.12 | Metastasis | OR (95% CI) | AA: 10.8, (1.40‚82.7), p=0.02 | --------------- |
| Habieb et al., 2021 | *BCHE* | RS1803274 | Missense | 3q26.1 | T | 0.2 | Metastasis | Not reported | p=<0.001 | --------------- |
| Zuo et al., 2020 | *CASC16* | RS4784227 | Intron | 16q12.1-q12.2 | T | 0.23 | Lymph Node Metastasis | OR (95% CI) | TC + TT: 1.41 (1.04-1.93), p=0.028 | --------------- |
|  |  | RS12922061 | Intron | 16q12.1-q12.2 | T | 0.23 | Lymph Node Metastasis | OR (95% CI) | T: 1.30 (1.02-1.65), p=0.034 | --------------- |
| Hu et al., 2018 | *CCL4* | RS10491121 | 2KB Upstream | 17q12 | A | 0.4 | Lymph Node Metastasis | OR (95% CI) | Luminal A + Luminal B:  AG: 0.298 (0.1-0.885) AG + GG vs. AA: 0.106 (0.011-1.038) | --------------- |
| Shaker et al., 2019 | CHI3L1 | RS4950928 | 2KB Upstream | 1q32.1 | G | 0.29 | Other Organ Metastasis | OR (95% CI) | --------------- | TCTCTG: 0.03 (0.004-0.24), p= < .0001 TGTCTG: 0.05 (.007-0.48), p= < .0001 |
|  | VDR | RS2228570 | Initiator Codon | 12q13.11 | A | 0.39 | Other Organ Metastasis | OR (95% CI) | --------------- | TCTCTG: 0.03 (0.004-0.24), p= < .0001 TGTCTG: 0.05 (.007-0.48), p= < .0001 |
|  |  | RS1544410 | Intron | 12q13.11 | T | 0.4 | Other Organ Metastasis | OR (95% CI) | --------------- | TCTCTG: 0.03 (0.004-0.24), p= < .0001 TGTCTG: 0.05 (.007-0.48), p= < .0001 |
|  | RANKL | RS9533156 | Intron | 13q14.11 | C | 0.45 | Other Organ Metastasis | OR (95% CI) | --------------- | TCTCTG: 0.03 (0.004-0.24), p= < .0001 TGTCTG: 0.05 (.007-0.48), p= < .0001 |
|  | OPG | RS2073618 | Missense | 8q24.12 | C | 0.50 | Other Organ Metastasis | OR (95% CI) | --------------- | TCTCTG: 0.03 (0.004-0.24), p= < .0001 TGTCTG: 0.05 (.007-0.48), p= < .0001 |
|  |  | RS2073617 | 2KB Upstream | 8q24.12 | A | 0.49 | Other Organ Metastasis | OR (95% CI) | --------------- | TCTCTG: 0.03 (0.004-0.24), p= < .0001 TGTCTG: 0.05 (.007-0.48), p= < .0001 |
| Saadawy et al., 2023 | *Circ-ITCH* | RS10485505 | Intron | 20q11.22 | T | 0.06 | Lymph Node Metastasis | Not reported | p=<0.001 | --------------- |
|  |  | RS4911154 | Intron | 20q11.22 | A | 0.16 | Lymph Node Metastasis | Not reported | p=0.0016 | --------------- |
| Babteen et al., 2020 | *CTLA4* | RS231775 | Missense | 2q33.2 | G | 0.37 | Metastasis | OR (95% CI) | 4.46 (1.18-16.87), p=0.019 | --------------- |
| Guembarovski et al., 2018 | *CXCL12* | RS1801157 | 3’ UTR | 10q11.21 | T | 0.18 | Lymph Node Metastasis | Tau-b correlation coefficient | -0.478, p=0.036 | --------------- |
| Martinez-Ramirez et al., 2021 | *CYP1B1* | RS1056836 | Missense | 2p22.2 | C | 0.43 | Lymph Node Status | OR (95% CI) | C/G: 0.50 (0.24-1.05); p=0.04 | --------------- |
| Mei et al., 2019 | *DAAM1* | RS79036859 | 3’ UTR | 14q23.1 | C | 0.0 | Lymph Node Metastasis | OR (95% CI) | 3.90 (1.77-8.59), p=<0.001 | --------------- |
| Bidkani et al., 2018 | *ERBB4* | RS13423759 | 3’ UTR | 2q34 | C | 0.04 | Metastasis | OR (95% CI) | 2.893 (1.128-7.422), p=0.025 | --------------- |
| Carrillo-Morento et al., 2019 | *ESR1* | RS9340799 | Intron | 6q25.1-q25.2 | G | 0.34 | Lymph Node Metastasis | OR (95% CI) | GG: 2.85 (1.144-7.10), p=0.024 | --------------- |
| Gallegos-Arreola et al., 2022 | *ESR2* | RS1256030 | Intron | 14q23.2-q23.3 | A | 0.44 | Lymph Node Metastasis | OR (95% CI) | TT: 2.67 (1.29-5.53), p=0.010  HER2, TT: 0.38 (0.18-0.78), p=0.005 | --------------- |
| Wu et al., 2019 | *GALNT16* | RS2105269 | Intron | 14q24.1 | G | 0.31 | Lymph Node Metastasis | OR (95% CI) | heterozygote model: 1.59 (1.10-2.32) | --------------- |
| Fesharaki et al., 2020 | *GAPDH* | RS1803622 | Non-Coding Transcript | 12p31.31 | T | 0.27 | Metastasis | OR (value not reported) p-value | p=0.00039 (frequency of alleles breast cancer samples v. controls)  p=0.005 (distribution of genotype in breast cancer samples v. controls) | --------------- |
| Mir et al., 2021 | *HIF-1a* | RS11549465 | Missense | 14q23.2 | T | 0.11 | Distant Metastasis | Chi-sq | 9.48, p=0.008 | --------------- |
| Padala et al., 2022 | *IL-6* | RS1800795 | 2KB Upstream | 7p15.3 | C | 0.36 | Lymph Node Status | OR (95% CI) | --------------- | IL-6 haplotype (RS1800795, RS1800796, RS1800797) ACC: 0.52 (0.29-0.92), p=0.025 |
|  |  | RS1800796 | 2KB Upstream | 7p15.3 | C | 0.07 | Lymph Node Status | OR (95% CI) | --------------- | IL-6 haplotype (RS1800795, RS1800796, RS1800797) ACC: 0.52 (0.29-0.92), p=0.025 |
|  |  | RS1800797 | 2KB Upstream | 7p15.3 | A | 0.36 | Lymph Node Status | OR (95% CI) | --------------- | IL-6 haplotype (RS1800795, RS1800796, RS1800797) ACC: 0.52 (0.29-0.92), p=0.025 |
| Al-Ankoshy et al., 2019 | *IL-10* | RS1800896 | 2KB Upstream | 1q32.1 | C | 0.45 | Metastasis | OR (95% CI) | 3.3(1.2-7.8), p=0.006 | --------------- |
| Padala et al., 2022 | *IL-18* | RS187238 | 2KB Upstream | 11q23.1 | C | 0.14 | Metastasis | OR (95% CI) | GG: 2.41 (1.43-4.07) CC: 2.85 (1.32‚6.15) p=0.001 | --------------- |
|  |  |  | 2KB Upstream | 11q23.1 | C | 0.14 | Lymph Node Status | OR (95% CI) | --------------- | IL-18 haplotype (RS1946518, RS187238, RS549908) CCA: 2.00 (1.08-3.70), p=0.001 |
|  |  | RS1946518 | 2KB Upstream | 11q23.1 | T | 0.41 | Lymph Node Status | OR (95% CI) | --------------- | IL-18 haplotype (RS1946518, RS187238, RS549908) CCA: p=0.027 |
|  |  | RS549908 | Synonymous | 11q23.1 | G | 0.30 | Lymph Node Status | OR (95% CI) | --------------- | IL-18 haplotype (RS1946518, RS187238, RS549908) CCA: p=0.027 |
| He et al., 2021 | *IL-2RB* | RS2281089 | Intron | 22q12.3 | G | 0.16 | Metastasis | OR (95% CI) | A: 0.72 (0.55-0.95), p=0.022 dom: 0.69 (0.49-0.97), p=0.035 log-add: 0.72 (0.55-0.96), p=0.023 | --------------- |
| Vitiello et al., 2018 | *IL7RA* | RS6897932 | Missense | 5p13.2 | T | 0.25 | Lymph Node Metastasis | Tau-b correlation coefficient | L-HER2+ add: 0.32; p=0.03 dom: 0.35; p=0.02 | --------------- |
| Gallegos-Arreola et al., 2021 | *KRAS* | RS61764370 | 3’ UTR | 12p12.1 | C | 0.07 | Lymph Node Metastasis | OR (95% CI) | HER2: 3.4 (1.24-9.84), p=0.018 | --------------- |
| Gallegos-Arreola et al., 2020 | *KRAS* | RS712 | 3’ UTR | 12p12.1 | C | 0.50 | Lymph Node Metastasis | OR (95% CI) | Luminal B (genotype G/T): 0.241 (0.093-0.626), p=0.003 | --------------- |
| Shan et al., 2019 | *MAP3K21* | RS1294255 | Intron | 1q42.2 | G | 0.37 | Lymph Node Invasion | OR (95% CI) | GC v. GG: 0.47 (0.29-0.74), p=0.001 | --------------- |
| Martinez-Ramirez et al., 2021 | *MGMT* | RS12917 | Missense | 10q26.3 | T | 0.13 | Lymph Node Status | OR (95% CI) | C/T: 1.75 (0.77-3.95), p=0.03 | --------------- |
| Iranparast et al., 2023 | *miRNA-155* | RS767649 | 2KB Upstream | 21q21.3 | A | 0.07 | Lymph Node Metastasis | Correlation coefficient | alleles (with A allele v. without A allele)  r=0.261, p=0.001  genotypes (AA, AT, TT)  r=0.292, p=0.001 | --------------- |
| Mir et al., 2018 | *miRNA-423* | RS6505162 | Non-coding transcript | 17q11.2 | C | 0.46 | Distant Metastasis | Chi-sq | 9.25, p=0.009 | --------------- |
| Habel et al., 2019 | *MMP-2* | RS2285053 | 2KB Upstream | 16q12.2 | T | 0.12 | Distant Metastasis | Spearmans correlation coefficient | p=0.001 | --------------- |
|  |  | RS243866 | 2KB Upstream | 16q12.2 | G | 0.23 | Nodal Status | Spearmans correlation coefficient | p=0.030 | --------------- |
| Manshadi et al., 2018 | *MMP-9* | RS3918242 | 2KB Upstream | 20q13.12 | T | 0.17 | Metastasis | Not reported | p=0.029 | --------------- |
| Pirooz et al., 2018 | *MMP-9* | RS1056628 | 3’ UTR | 20q13.12 | C | 0 | Metastasis | OR (95% CI) | 2.23 (1.03-6.9) | --------------- |
| Ghanei et al., 2018 | *NBS1* | RS2735383 | 3’ UTR | 8q21.3 | C | 0.33 | Lymph Node Metastasis | OR (95% CI) | 0.23 (0.11-0.52), p = < 0.001 | --------------- |
| Ghali et al., 2019 | *NF-KB* | RS148626207 | Missense | 4q24 | C | 0 | Distant Metastasis | Not reported | p=0.006 | --------------- |
|  |  | RS3774937 | Intron | 4p24 | C | 0.29 | Distant Metastasis | Not reported | p=0.039 | --------------- |
| Antar et al., 2020 | *NME1* | RS34214448 | Intron | 17q21.33 | T | 0.4 | Distant Metastasis | OR (95% CI) | G/T: 0.25 (0.071-0.884), p=0.031 | --------------- |
| Wei et al., 2020 | *NR5A2* | RS2246209 | 3’ UTR | 1q32.1 | G | 0.35 | Lymph Node Metastasis | OR (95% CI) | dom: 0.65 (0.46-0.94), p=0.021 log-add: 0.70 (0.53-0.93), p=0.015 | --------------- |
| Hayat et al., 2022 | *OPG* | RS3102735 | 2KB Upstream | 8q24.12 | C | 0.15 | Metastasis | OR (95% CI) | TC: 0.1704 (0.0716-0.4053), p=0.001  CC: 0.0517 (0.0157-0.1708), p=0.0001  C: 0.1704 (0.0716-0.4053), p=0.0001 | --------------- |
| Hayat et al., 2022 | *RANKL* | RS9533156 | Intron | 13q14.11 | C | 0.45 | Metastasis | OR (95% CI) | C: 0.5177(0.2994-0.8953), p=0.0185 | --------------- |
| Cai et al., 2019 | *SENP2* | RS6762208 | Missense | 3q27.2 | C | 0.35 | Lymph Node Status | OR (95% CI) | AA: 3.223 (2.068-5.563), p=0.034  CA+AA: 3.218 (2.312-7.898), p=0.011  A: 2.870 (1.370-4.010), p=0.005 | --------------- |
| Gallegos-Arreola et al., 2020 | *SOD1* | RS2234694 | Intron | 21q22.11 | C | 0.04 | Lymph Node Metastasis | OR (95% CI) | 1.5 (1.1-2.25), p=0.019 | A + C: 2.37 (1.08-5.19), p=0.031 |
| Gallegos-Arreola et al., 2023 | *SOD1* | RS4817415 | None | 21q22.11 | A | 0.29 | Lymph Node Metastasis | OR (95% CI) | CC: 3.2 (1.33-7.7). p=0.014 | --------------- |
| Asadi et al., 2022 | *SOD2* | RS2758339 | Intron | 6q25.3 | C | 0.43 | Other Organ Metastasis | OR (95% CI) | C: 4.528 (0.985–20.80), p=0.046 dom: 0.190 (0.034–1.039), p=0.049 | --------------- |
| Mohammadi et al., 2022 | *TCF3* | RS72618599 | Intron | 19p13.3 | T | 0.01 | Metastasis | Not reported | p=0.000 (# of genotype) p=0.000 (# of allele) | --------------- |
| Hadj-Ahmed et al., 2019 | *TGFB1* | RS1800469 | 2KB Upstream | 19q13.2 | A | 0.33 | Distant Metastasis | Pearson's correlation coefficient | p=3.5 x 10^-4 | --------------- |
|  |  |  | 2KB Upstream | 19q13.2 | A | 0.33 | Lymph Node Status | Kendall's Tau-b rank correlation | rs1800469: p=0.001 | --------------- |
|  |  | RS1800470 | Missense | 19q13.2 | G | 0.5 | Lymph Node Status | Pearson's correlation coefficient | rs1800470: p=0.038 | --------------- |
| Vitiello et al., 2018 | *TGFB1* | RS1800469 | 2KB Upstream | 19q13.2 | A | 0.33 | Lymph Node Metastasis | Kendall's Tau-b rank correlation | HER2+ rec: 0.34**  TN genotypic: 0.29*  *p < 0.05 **p < 0.001 | HER2+  (RS1800469, RS1800470) GTCG (rec): 0.38**  TN  (RS1800469, RS1800470) GCTG (rec): -0.32*  *p < 0.05 **p < 0.001 |
|  |  | RS1800470 | Missense | 19q13.2 | G | 0.5 | Lymph Node Metastasis | Kendall's Tau-b rank correlation | HER2+ dom: -0.31*  *p < 0.05 **p < 0.001 | HER2+  (RS1800469, RS1800470) GTCG (rec): 0.38**  *p < 0.05 **p < 0.001 |
| Albalawi et al., 2020 | *VEGFA* | RS833061 | 2KB Upstream | 6p21.1 | C | 0.48 | Distant Metastasis | Chi-sq | CC v. CT v. TT = 13.88, p= 0.001 | --------------- |
| **Cohort** | | | | | | | | | | |
| Korobeinikova et al., 2021 | *ATF3* | RS3125289 | Intron | 1q32.3 | T | 0.49 | Distant Metastasis | OR (95% CI) | TT: 0.116 (0.015-0.911), p=0.040 | --------------- |
|  |  |  |  |  |  |  | Lymph Node Status | OR (95% CI) | TT: 0.336 (0.115-0.980), p=0.046 | --------------- |
|  |  | RS11119982 | Intron | 1q32.3 | T | 0.47 | Distant Metastasis | OR (95% CI) | TT: 0.194 (0.052-0.726), p=0.015 TC+TT: 0.367 (0.162-0.833), p=0.017 | --------------- |
| Bekampyte et al., 2021 | *BBC3* | RS2032809 | 2KB Upstream | 19q13.32 | T | 0.41 | Metastasis | OR (95% CI) | AG v. AA: 4.246, 95% CI (1.184-15.222), p=0.026 | --------------- |
| Sun et al., 2020 | *CASC16* | RS4784227 | Intron | 16q12.1-q12.2 | T | 0.23 | Lymph Node Metastasis | OR (95% CI) | T: 1.51 (1.05-2.17), p=0.025 co-dom C/T: 1.99 (1.20-3.31), p=0.008 dom C/T-T/T: 1.94 (1.19-3.16), p=0.008 log-add: 1.52 (1.06-2.19), p=0.023 | --------------- |
| Huang et al., 2018 | *HMGB1* | RS1360485 | 3’ UTR | 13q12.3 | C | 0.30 | Lymph Node Metastasis | OR (95% CI) | 1.444 (0.944-2.207)  *p-value not reported, but authors indicated it was <0.05 | --------------- |
|  |  | RS1045411 | 3’ UTR | 13q12.3 | T | 0.23 | Lymph Node Metastasis | OR (95% CI) | 1.443 (0.935-2.228)  *p-value not reported, but authors indicated it was <0.05 | --------------- |
|  |  | RS2249825 | Intron | 13q12.3 | C | 0.15 | Lymph Node Metastasis | OR (95% CI) | 1.515 (0.937-2.448)  *p-value not reported, but authors indicated it was <0.05 | --------------- |
| Korobeinikova et al., 2021 | *P21* | RS1801270 | Missense | 6p21.2 | A | 0.09 | Lymph Node Metastasis | OR (95% CI) | CA+AA: 0.426 (0.149, 0.865), p=0.041 | --------------- |
| Golubickaite et al., 2018 | *POLG* | RS2072267 | Intron | 15q26.1 | G | 0.47 | Progression | OR (95% CI) | AG vs. GG: model 1: 3.386 (1.262-9.086), p=0.015 model 2: 4.552 (1.531-13.536), p=0.006 model 3: 4.243 (1.427-12.614), p=0.009 | --------------- |
|  |  | RS2307441 | Missense | 15q26.1 | C | 0.04 | Vascular Invasion | OR (95% CI) | TT vs. CC: model 1: 0.075 (0.017-0.323), p=0.001 model 2: 0.064 (0.014-0.289), p=0.000 model 3: 0.008n (0.001-0.055), p=0.000  TC vs. CC: model 1:0.032 (0.006-0.168), p=0.000 model 2: 0.027 (0.005-0.151), p=0.000 model 3:0.002 (0.000-0.021), p=0.000 | --------------- |
| Golubickaite et al., 2018 | *TFAM* | RS3900887 | Intron | 10q21.1 | A | 0.15 | Lymph Node Involvement | OR (95% CI) | TT vs. AA: model 1: 6.286 (2.285-17.289), p=0.000 model 2: 5.556 (1.982-15.572), p=0.001 model 3: 4.708 (1.699-13.047), p=0.003  TA vs. AA: model 1: 7.527 (2.202-25.729), p=0.001 model 2: 6.839 (1.967-23.780), p=0.002 model 3: 5.900 (1.718-20.254), p=0.005 | --------------- |
|  |  |  |  |  |  |  | Lymphatic Invasion | OR (95% CI) | TT vs. AA: model 1: 6.781 (2.138-21.503), p=0.001 model 2: 4.614 (1.382-15.402), p=0.013 model 3: 10.526 (2.446-45.292), p=0.002 | --------------- |
|  |  | RS11006129 | Intron | 10q21.1 | T | 0.15 | Lymph Node Involvement | OR (95% CI) | CC vs. TT: model 1: 3.583 (1.044-12.295), p=0.042 | --------------- |
|  |  |  |  |  |  |  | Vascular Invasion | OR (95% CI) | CT vs. TT:  model 1: 0.160 (0.039-0.652), p=0.011 | --------------- |
| Liu et al., 2020 | *TIMP-2* | RS4789936 | Intron | 17q25.3 | C | 0.49 | Lymph Node Metastasis | HR (95% CI) | Univariate analysis: 2.35 (1.05-5.28), p=0.038 Multivariate analysis: 2.85 (1.03-7.83), p=0.043 | --------------- |
| Korobeinikova et al., 2021 | *TXNRD2* | RS1139793 | Missense | 22q11.21 | A | 0.25 | Distant Metastasis | OR (95% CI) | GA+AA: 0.421 (0.184-0.964), p=0.041 | --------------- |
